# Editorial: Women in comparative immunology

**DOI:** 10.3389/fimmu.2023.1358322

**Published:** 2024-01-09

**Authors:** Annalisa Pinsino, Noemí Sevilla

**Affiliations:** ^1^ Institute of Translational Pharmacology (IFT), National Research Council (CNR), Palermo, Italy; ^2^ Centro de Investigación en Sanidad Animal, Instituto Nacional de Investigación y Tecnología Agraria y Alimentaria, Consejo Superior de Investigaciones Científicas (CISA-INIA-CSIC), Madrid, Spain

**Keywords:** immune response, model organisms, sea urchin, horse, pig, high-throughput technologies, women scientists

Comparative immunology researches the similarities and differences among living organisms merging physiological, functional, and genomic information to understand the evolution of the immune response. The diversity of defensive solutions hired by organisms to control pathogens, chemical contaminants, and malignant cell transformation seems boundless because of the capability of organisms to adapt to their environment and acquire resistance to infections and diseases. As a discipline, comparative immunology began in the sixties when many immunologists pursued a comparative approach to immunology to obtain information on those aspects of immunity that are universal because they are phylogenetically preserved. Progress in the field was hampered for a long time by the resistance of scientists whose thinking was entrenched in the idea of the apparent problems of successfully translating these results into human immunity (homocentric vision and scientific prejudice). Today comparative immunology is progressing fast and the development of high-throughput technologies such as deep-sequencing and bioinformatics has opened new frontiers to explore the diversity of immunity in model organisms for a better understanding of human beings. This has involved the discovery of new biomolecules and functions that further knowledge of immunological evolution and provide new solutions to mitigate human diseases. Many women have been at the forefront of this progress and in recent years scientists have conducted groundbreaking research in immunology, contributing to our understanding of the immune system in various species, including humans and animals. This Research Topic aims to highlight research across all these possible fields of comparative immunology, promoting work led by women scientists. Barela Hudgell and Smith, Yakovenko et al., Simonin et al., Wiarda and Loving have contributed to the growth of this SI, publishing their most recent results in the field.

Barela Hudgell and Smith, Yakovenko et al. contribute to understanding of the immune system in a marine invertebrate model, the sea urchin. This echinoderm has received great attention as an unexploited source of functional molecules and unpredicted solutions of defenses involved in immunity. The sea urchins feel environmental stimuli through an abundant and expanded repertoire of genes that encode proteins with predicted immune function identified based on homology at the RNA level (1). The SpTransformer proteins (*Sp*Trf*;* formerly Sp185/333) of the sea urchin *Strongylocentrotes purpuratus* — studied by the L. Courtney Smith Team for over fifteen years — are an example of the huge level of diversity and selectivity of the sea urchin innate immune response. The *SpTrf* gene family is involved in defensive functions against bacterial infections. Each specimen of sea urchin can express many *Sp*Trf protein variants as the product of a long-standing evolutionary host-pathogen arms race. Genes occur as mosaics of defined element patterns leading to interesting issues concerning the evolution of this gene family probably resulting from deletions, duplications, and gene conversion in the coding regions. Barela Hudgell and Smith have recently highlighted that although the manual alignments are essential to identify elements of these peculiar proteins, the computational approaches, including the new phylogeny-aware multiple sequence alignment program (PRANK), which makes use of evolutionary data to help place insertions and deletions, can help in detecting alternative alignments. Instead, Yakovenko et al. have shown that the recombination activating genes, RAG1 and RAG2, are the leading mediators of the V(D)J recombination of the antibodies and T-cell receptor molecules in vertebrates and are co-expressed in sea urchin embryos and adult tissues (upper digestive system, immune cells). Notably, one immunological target for RAG1L is the *Trf* gene family, endangered for a somatic gene diversification in sea urchin immune cells (2).

In the field of immunology, allergies are undoubtedly one of the most enigmatic responses within the area. The production of immunoglobulin E (IgE) that recognizes an allergen is certainly one of the key markers indicating an allergic process. However, there are still no identified markers that indicate whether an immunotherapy treatment for developed allergies is effective. Therefore, it is necessary to conduct research in this area to obtain a reliable biomarker that allows us to provide an accurate diagnosis by using animal models of allergy. Simonin et al. have invested in this field of research. The model of Culicoides hypersensitivity mediated by an IgE response and developed by horses in response to Culicoides saliva has allowed them to study the relationship between this allergen and the migration of IgE+ plasmablast cells to the periphery. The study has revealed that IgE+ plasmablasts serve as sensitive indicators of allergen exposure, entering the peripheral blood almost immediately after the presence of the allergen in the environment. Results suggest that these IgE+ plasmablasts can be considered good candidates as markers to assess the success of allergen immunotherapy treatment.

Intestinal intraepithelial lymphocytes (IELs) are integral components of the immune system in the gastrointestinal tract, playing key roles in immune surveillance, barrier function, tolerance, response to infections, inflammatory processes, and tissue repair. Understanding their functions is essential for comprehending the overall immune response in the gut and for developing strategies to manage gastrointestinal diseases. The relevance of IELs varies across different species, reflecting the diversity of immune system adaptations to the specific needs and challenges of each organism. Pigs have been used as models for studying the gastrointestinal immune system due to similarities in anatomy and physiology with humans. IELs in pigs contribute to mucosal defense and immune regulation and understanding porcine IELs is relevant for improving swine health and addressing challenges in pig farming. Wiarda and Loving provide an elegant and comprehensive review of current knowledge of IELs in pigs. The similarities between porcine and human digestive systems make pigs valuable models for studying intestinal health. The insights gained from pig IEL research may have direct implications for understanding human intestinal immune responses. If findings related to porcine IELs can be applied to humans, it could open new avenues for research and therapeutic development in the context of intestinal health and immune function.

## Author contributions

AP: Writing – original draft, Writing – review & editing. NS: Writing – original draft, Writing – review & editing.
